# MicroRNA-410 acts as oncogene in NSCLC through downregulating SLC34A2 *via* activating Wnt/β-catenin pathway

**DOI:** 10.18632/oncotarget.7538

**Published:** 2016-02-20

**Authors:** Xuechao Zhang, Xixian Ke, Qiang Pu, Yue Yuan, Weihan Yang, Xinmei Luo, Qianqian Jiang, Xueting Hu, Yi Gong, Kui Tang, Xiaolan Su, Lunxu Liu, Wen Zhu, Yuquan Wei

**Affiliations:** ^1^ State Key Laboratory of Biotherapy/Collaborative Innovation Center of Biotherapy, West China Hospital, Sichuan University, Chengdu, Sichuan Province, People's Republic of China; ^2^ Department of Thoracic Surgery, West China Hospital, Sichuan University, Chengdu, Sichuan Province, People's Republic of China

**Keywords:** miR-410, SLC34A2, NSCLC, tumorigenesis and development

## Abstract

*SLC34A2* had been reported to be down-regulated in human NSCLC cells and patient tissues, and played a significant role in lung cancer. However, the mechanism of its unusual expressionin NSCLC has not been fully elucidated. In present study, we identified *SLC34A2* was a direct target of miR-410 and could be inhibited by miR-410 transcriptionally and post-transcriptionally. MiR-410 promoted the growth, invasion and migration of NSCLC cells *in vitro*. An orthotopic xenograft nude mouse model further affirmed that miR-410 promoted NSCLC cell growth and metastasis *in vivo*. Moreover, restoring *SLC34A2* expression effectively reversed the miR-410-mediated promotion of cell growth, invasion and migration in NSCLC cells. In addition, miR-410^high^ /*SLC34A2*^low^ expression signature frequently existed in NSCLC cells and tumor tissues. MiR-410 significantly increased the expression of *DVL2* and *β-catenin* protein while decreased that of *Gsk3β* protein of Wnt/*β-catenin* signaling pathway, while *SLC34A2* partly blocked the effects of miR-410 on those protein expressions. Hence, our data for the first time delineated that unusual expression of *SLC34A2* was modulated by miR-410, and miR-410 might positivelycontribute to the tumorigenesis and development of NSCLC by down-regulating *SLC34A2* and activating Wnt/*β-catenin* signaling pathway. MiR-410 might be a new potential therapeutic target for NSCLC.

## INTRODUCTION

Lung cancer remains the world's most significant reason of cancer death and the mortality rate is still increasing [1]. Therefore, it is extremely important to elaborate the molecular mechanism of lung cancer pathogenesis and development. *SLC34A2* encoding NaPi2b plays an important role in the maintenance of the overall phosphate homeostasis which is essential for proper cellular functions such as DNA synthesis, cell signaling, bone formation etc. [2, 3]. *SLC34A2* is a tissue-specific transporter that is highly expressed in the lung [4-8]. In human lung, *SLC34A2* expresses only in Type II alveolar epithelium cells (AT-II) and is required for the synthesis of AT-II pulmonary surfactant [9-10]. AT-II cells are potential stem cells of the alveolar epithelium [11]. Increasing studies reported that AT-II cells might be transformed into cancer stem cells under exogenous or endogenous factors and induced carcinogenesis and development of NSCLC finally [11-14]. These indicated that *SLC34A2* might function physiologically in AT-II and its mutations or abnormal expression was bound to affect the normal function of AT-II which was related to lung tumorigenesis. Moreover, recent studies reported that *SLC34A2* played a critical role in lung cancer. Kopantzev et al. revealed expression of *SLC34A2* increased during the development of fetal lung and early embryonic development, but decreased in non-small cell lung carcinomas tissues compared with surrounding normal lung tissues [15]. Also, our lab previously reported that *SLC34A2* was down-regulated in human NSCLC tumor tissues and cells, and might act as tumor suppressor by inhibiting the growth, invasion and migration of lung cancer cells through the PI3K-Akt-mTOR and Ras-Raf-MEK-ERK signaling pathway [16, 17]. However, the mechanism of unusual expression *of SLC34A2* in NSCLC has not been fully elucidated. Therefore, it is of great significance to reveal the molecular mechanism of abnormal expression of *SLC34A2* for understanding the pathogenesis of NSCLC.

MicroRNAs (miRNAs), a family of small noncoding single-stranded RNAs, have been shown to play important roles in cancer cells and are tightly associated with the abnormal expression of tumor-relevant genes recently [18]. MiRNA leads to transcriptional silencing of gene expression through complementary pairing in 3′ UTR of its target mRNA. Recent studies acknowledged that more than 200 miRNAs regulating tumor-related genes expression were closely related to tumor development [19]. As one of the most deadly cancers, lung cancer was regulated by many miRNAs [20]. Dozens of miRNAs, such as miR-21, miR-17-92, miR-143/145, miR-34, miR-200, etc. played essential roles in lung tumorigenesis by regulating critical oncogene or tumor suppressor [21-25]. In present study, we aimed to identify a specific miRNA targeting *SLC34A2* for unclosing the mechanism of aberrant expression of *SLC34A2,* then further explored its function to the pathogenesis and development of NSCLC. We firstly demonstrated that *SLC34A2* was a direct target of miR-410 and inhibited by miR-410 transcriptionally and post-transcriptionally, and overexpression of miR-410 significantly promoted cell growth, invasion and metastasis by down-regulating *SLC34A2* via activating Wnt/*β- catenin* pathway. Hence, our study identified a new miRNA and signaling pathway for understanding the pathogenesis and provided promising therapeutic target for NSCLC.

## RESULTS

### SLC34A2 was identified as a direct target of miR-410

**Figure 1:**
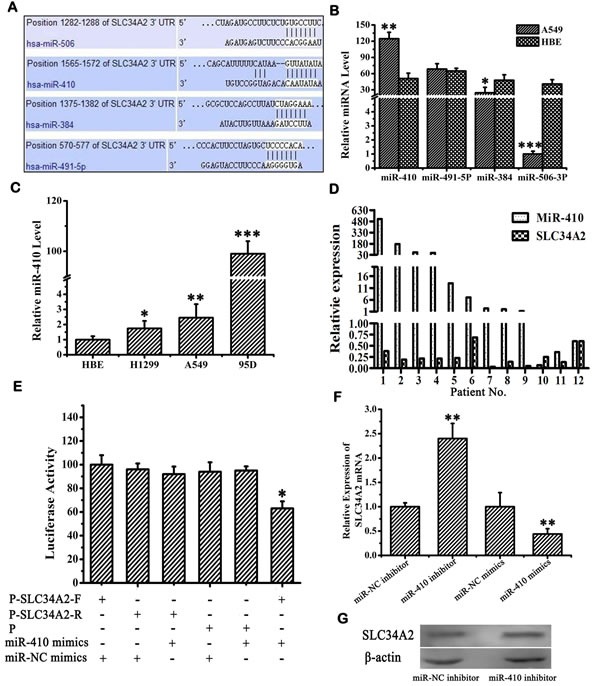
MiR-410 directly targeted SLC34A2 **A.** Four miRNAs (miR-410, miR-491-5P, miR-384 and miR-506-3P) were predicted by both algorithms (TargetScan, miRanda). The numbers indicate the nucleotide position in reference to the start of *SLC34A2* 3′UTR. **B.** The expression of miR-410, miR-491-5P, miR-384 and miR-506-3P in A549 cells was determined by qRT-PCR. **C.** The expressions of miR-410 in A549, 95D and H1299 cells were determined by qRT-PCR. **D.** Relative expression of miR-410 and *SLC34A2* detected by qRT-PCR in NSCLC patient tissues. Increased miR-410 expression and decreased *SLC34A2* expression were indicated in 9 of 12 NSCLC patient tissues compared with adjacent non-tumorous tissues. **E.** Luciferase reporter assay was performed to confirm the miR-410 binding to the 3′UTR of *SLC34A2*. The luciferase activity was detected after co-transfection with luciferase reporter plasmids (Pmir-*SLC34A2* 3′UTR-F, P-SLC34A2-F; Pmir-*SLC34A2* 3′UTR-R, P-SLC34A2-R), with miR-410 mimics/NC or miR-410 inhibitors/NC in HEK293 cells. **F.** Real-time PCR was performed to detect *SLC34A2* mRNA level after transfection of miR-410 inhibitors or miR-410 mimics with corresponding control in A549 cells. **G.** Western blotting was performed to detect *SLC34A2* protein level after transfection of miR-410 inhibitors with corresponding control in A549 cells. For miR-410 and *SLC34A2* mRNA expression detected by qRT-PCR, U6 and *β-actin* were used as internal control respectively. For *SLC34A2* protein expression detected by western blotting, *β-actin* was used as internal loading control. Data are presented as the mean value ± SD from triplicate experiments. *, *p* < 0.05; **, *p* < 0.01.

Two algorithms (TargetScan, miRanda) were used to predict miRNAs targeting *SLC34A2*. In light of individual computer-aided algorithms usually bringing about quantities of false positives, we applied a combination of two approaches to provide a more accurate assessment of the targeting miRNA. 22 miRNAs were preliminarily filtered (data not shown) and then four of them (miR-410, miR-506, miR-491, miR-384) were selected because of their lower free binding energy which meant more possibility that miRNA might bind to its target gene (Figure 1A). Next, we checked the expression of these four miRNAs by qRT-PCR in NSCLC cell line A549 in which *SLC34A2* was down-regulated compared with the normal cell line HBE. The expression of miR-410 was significantly up-regulated (*p* < 0.05), miR-491 displayed no expression change, miR-384 and miR-506 were both down-regulated respectively (*p* < 0.05) in A549 cells (Figure 1B). Since miR-410 was highly expressed in A549 cells, we further detected its expression in other NSCLC cell lines H1299 and 95D in which *SLC34A2* was also down-regulated compared with the normal cell line HBE. MiR-410 were significantly up-regualted in both cell lines compared with HBE (*p* < 0.05) (Figure 1C). Moreover, we found that miR-410 was significantly up-regulated and *SLC34A2* was significantly down-regulated in 9 of 12 NSCLC tumor tissues compared with adjacent non-tumorous tissues simultaneously by qRT-PCR (Figure 1D). These results indicated that overexpression of miR-410 might be associated with down-regulation of *SLC34A2*.

To further verify *SLC34A2* was a direct target of miR-410, 3′-UTR luciferase reporter plasmids containing the wild-type or mutant miR-410-binding sequences of *SLC34A2* were constructed and co-transfected with miR-410 mimics/NC into HEK-293 cells. The relative luciferase activity of the reporter containing wild-type Pmir-*SLC34A2* 3′UTR-F was significantly reduced when miR-410 mimics were co-transfected. In contrast, the luciferase activity of the reporter containing the mutant Pmir-*SLC34A2* 3′UTR-R was not affected by co-transfecting with miR-410 mimics, indicating that miR-410 might inhibit gene expression through miR-410-binding sequences at the 3′-UTR of *SLC34A2* (Figure 1E) (*p* < 0.05). We further analyzed the effects of miR-410 on *SLC34A2* expression in NSCLC cells. QRT-PCR and Western blotting were performed to check the effects of overexpression or inhibition of miR-410 on mRNA and protein expression levels of *SLC34A2* in A549 and 95D cell lines. *SLC34A2* mRNA was up-regulated or down-regulated when transfected with miR-410 inhibitors or miR-410 mimics into both A549 and 95D cell lines both A549 and 95D cell lines (Figure 1F) (*p* < 0.05). Similarly, *SLC34A2* protein level was up-regulated or down-regulated when transfected with miR-410 inhibitors or miR-410 mimics into both A549 and 95D cell lines (Figure 1G) (*p* < 0.05). These findings further confirmed that miR-410 could inhibit *SLC34A2* expression transcriptionally and post-transcriptionally.

### MiR-410 promoted proliferation, invasion and migration but inhibited apoptosis in NSCLC

**Figure 2:**
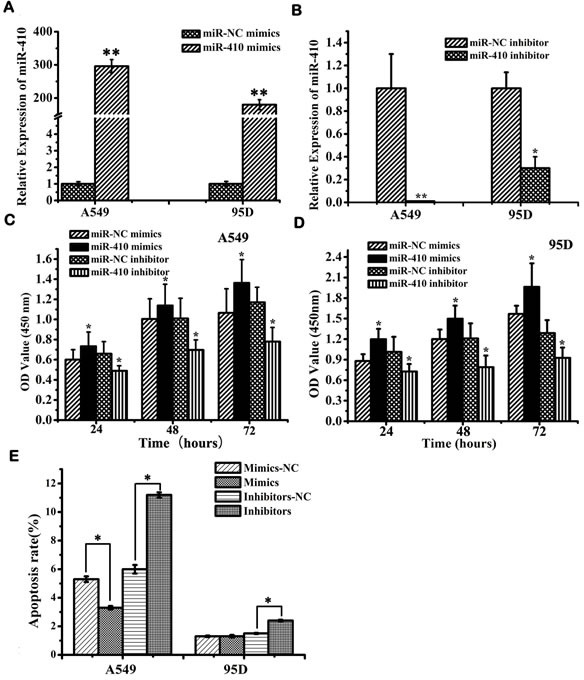
MiR-410 promoted cell proliferation and inhibited apoptosis of NSCLC cells **A.** and **B.** Real-time RT-PCR showed miR-410 expression in A549 and 95D cells after transfection of miR-410 inhibitors/NC **A.** or miR-410 mimics/NC **B.**. **C.** and **D.** MTT assay displayed that overexpression or inhibition of miR-410 promoted or prohibited cell growth of A549 **C.** and 95D **D.** cells. **E.** Overexpression or inhibition of miR-410 suppressed or enhanced apoptosis of A549 and 95D cells. Data are presented as the mean value ± SD from triplicate experiments. *, *p* < 0.05; **, *p* < 0.01.

**Figure 3:**
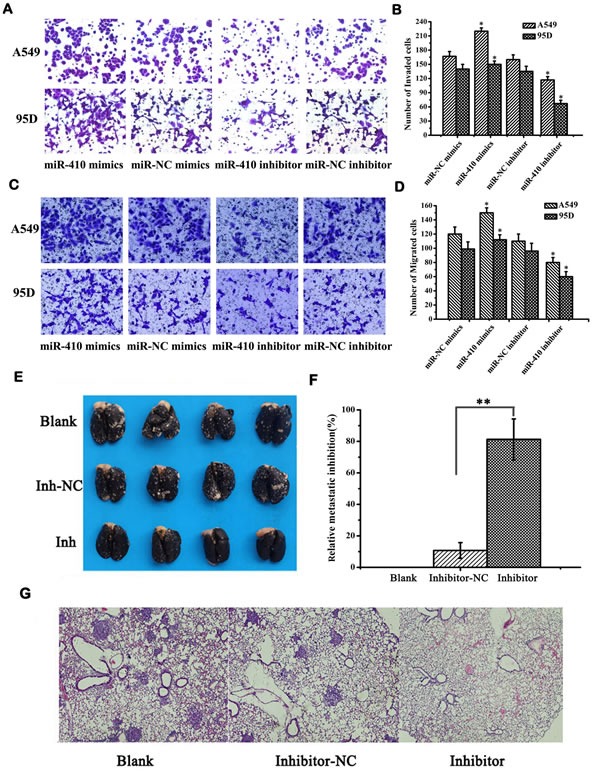
MiR-410 promoted invasion and migration of NSCLC cells *in vivo* and *in vitro* **A.** and **B.** Transwell assay showed overexpression or inhibition of miR-410 promoted or prohibited invasion of A549 and 95D cells (100×). **C.** and **D.** Millicell assay showed that overexpression or inhibition of miR-410 promoted or prohibited migration of A549 and 95D cells (100×). **E.** Metastatic tumors in the lungs. About 5 weeks later, mice were anesthetized and their lungs were filled with India ink to examine the number of metastasis nodules. **F.** Relative inhibition of metastasis of lymph node. The percent of metastatic inhibition was calculated by comparing with A549-treated blank control. **G.** Representative images of H&E staining of the lung tissues of mice (40×). Data are presented as the mean value ± SD from triplicate experiments. *, *p* < 0.05; **, *p* < 0.01.

Next, the *in vitro* effects of miR-410 abnormal expression on malignant phenotypes of NSCLC cells were investigated. The expression of miR-410 was significantly up-regulated or down-regulated after transfection with miR-410 mimics or inhibitors compared with respective NC in both A549 and 95D cell lines (Figure 2A and 2B) (*p* < 0.05). MTT assay showed cell growth was greatly enhanced after transfecting with miR-410 mimics compared with the matched NC in both A549 and 95D cells (Figure 2C and 2D) (*p* < 0.05). On the contrary, cell growth was greatly inhibited after transfection with miR-410 inhibitors compared with the NC in both A549 and 95D cells (Figure 2C and 2D) (*p* < 0.05). To explore the possible mechanism of miR-410 promoting cell growth, we performed apoptotic analysis. Apoptotic assay via FACS showed that apoptotic rate was reduced in cells transfected with miR-410 mimics than that of NC, while conversely, apoptotic rate was elevated in cells transfected with miR-410 inhibitors than that of NC (Figure 2E) (*p* < 0.05). These results suggested that miR-410 could promote cell proliferation and inhibited apoptosis of NSCLC cells *in vitro*. Next, we determined the *in vitro* effects of miR-410 on invasion and migration in A549 and 95D cells. By *in vitro* Transwell assay, we observed cell invasion were significantly impaired after transfecting with miR-410 inhibitors, and enhanced after transfecting with miR-410 mimics compared with respective scramble control in A549 and 95D cells (Figure 3A and 3B) (*p* < 0.05). *In vitro* Millicell assay displayed cell migration was impaired after transfecting with miR-410 inhibitors and strengthened after transfecting with miR-410 mimics compared with respective scramble control in both A549 and 95D cells (Figure 3C and 3D) (*p* < 0.05).

To further investigate the role of miR-410 on the growth and metastasis of NSCLC *in vivo*, we firstly established the miR-410 stable knockdown cells (INH-LV) and relevant scrambled control cells (INH-NC-LV) in A549 cell lines. Then, we injected these cells into mice via tail vain to build lung metastasis nude mouse model. About 10 weeks later, lungs of five mice in each group were injected intratracheally with India ink and fixed in AAF solution (85% ethanol, 10% acetic acid, 5% formalin) to count the number of metastatic tumor nodules on lung surfaces. We firstly observed that tumor metastasis nodules in the lungs of mice reduced by 71.6% in mice treated with miR-410 stable knockdown cells INH-LV compared with scrambled control cells INH-NC-LV (Figure 3E and 3F) (*p* < 0.01). And the metastasis nodules were also bigger in mice treated with miR-410 stable knockdown cells INH-LV than those of mice treated with scrambled control cells INH-NC-LV (Figure 3E). Consistently, H&E staining of lung tissue sections also displayed that the lungs of the mice treated with miR-410 stable knockdown A549 cells had much fewer nodules, and most of the lungs were out of tumor nodules (Figure 3G). These results confirmed that miR-410 promoted growth and metastasis of NSCLC both *in vitro* and *in vivo*.

### MiR-410 promoted proliferation, invasion and migration by down-regulating *SLC34A2* in NSCLC cells

**Figure 4:**
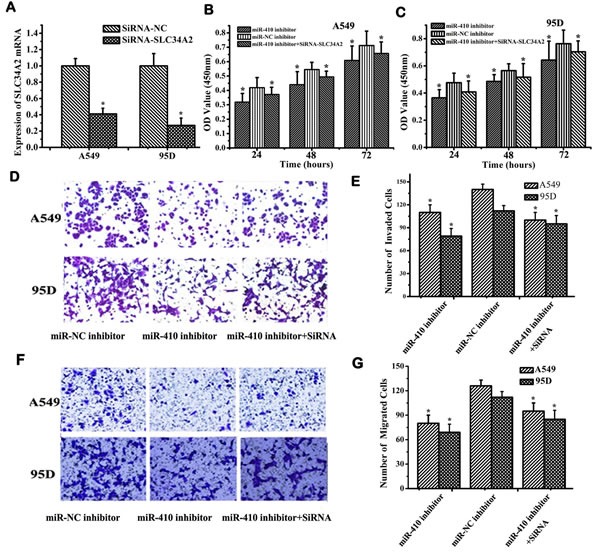
MiR-410 promoted proliferation, invasion and migration of NSCLC cells through targeting SLC34A2 *in vitro* **A.** QRT-PCR detection of *SLC34A2* mRNA in NSCLC A549 and 95D cells after transfecting with siRNA-*SLC34A2*/siRNA-NC. **B.** and **C.** MTT assay showed that inhibition of cell proliferation was partly restored in A549 **B.** and 95D **C.** cells after co-transfecting with siRNA-*SLC34A2* and miR-410 inhibitor compared with that of cells transfected with miR-410 inhibitor. Transwell **D.** and **E.** and Millicell **F.** and **G.** assay indicated that inhibition of cell invasion and migration was partially restored in A549 and 95D cells after co-transfecting with siRNA-*SLC34A2* and miR-410 inhibitor compared with that of cells transfected with miR-410 inhibitor (100×). Data are presented as the mean value ± SD from triplicate experiments. *, *p* < 0.05; **, *p* < 0.01.

To further investigate whether miR-410-activating cellular effects were mediated through down-regulation of *SLC34A2* in NSCLC cells, miR-410 inhibitors were transfected into A549 and 95D cells with or without siRNA-*SLC34A2* and subsequently scored for cell proliferation, migration and invasion. Firstly, *SLC34A2* mRNA was detected to check the knockdown efficiency of siRNA-*SLC34A2* in A549 and 95D cells before transfection. The expression levels of *SLC34A2* mRNA in siRNA-*SLC34A2*-transfected cells were significantly decreased compared with that of siRNA-NC-transfected cells (Figure 4A). Cell proliferation was inhibited in cells transfected with miR-410 inhibitors, while partly enhanced when cells were cotransfected with miR-410 inhibitors and siRNA-*SLC34A2* compared with their respective NC control via MTT assay (Figure 4B and 4C). Similarly, the effects on cell migration and invasion were also checked using Millicell and Transwell assay. Cell migration and invasion were inhibited in cells transfected with miR-410 inhibitors, while partly promoted when cells were cotransfected with miR-410 inhibitors and siRNA-*SLC34A2* compared with their respective NC control (Figure 4D, 4E and Figure 4F, 4G) (*p* < 0.05). These results proved that miR-410 promoted proliferation, invasion and migration in NSCLC cells partially dependent on down-regulation of *SLC34A2 in vitro*.

### Increased expression of miR-410 and reduced expression of *SLC34A2* frequently existed in NSCLC tumor tissues

**Figure 5:**
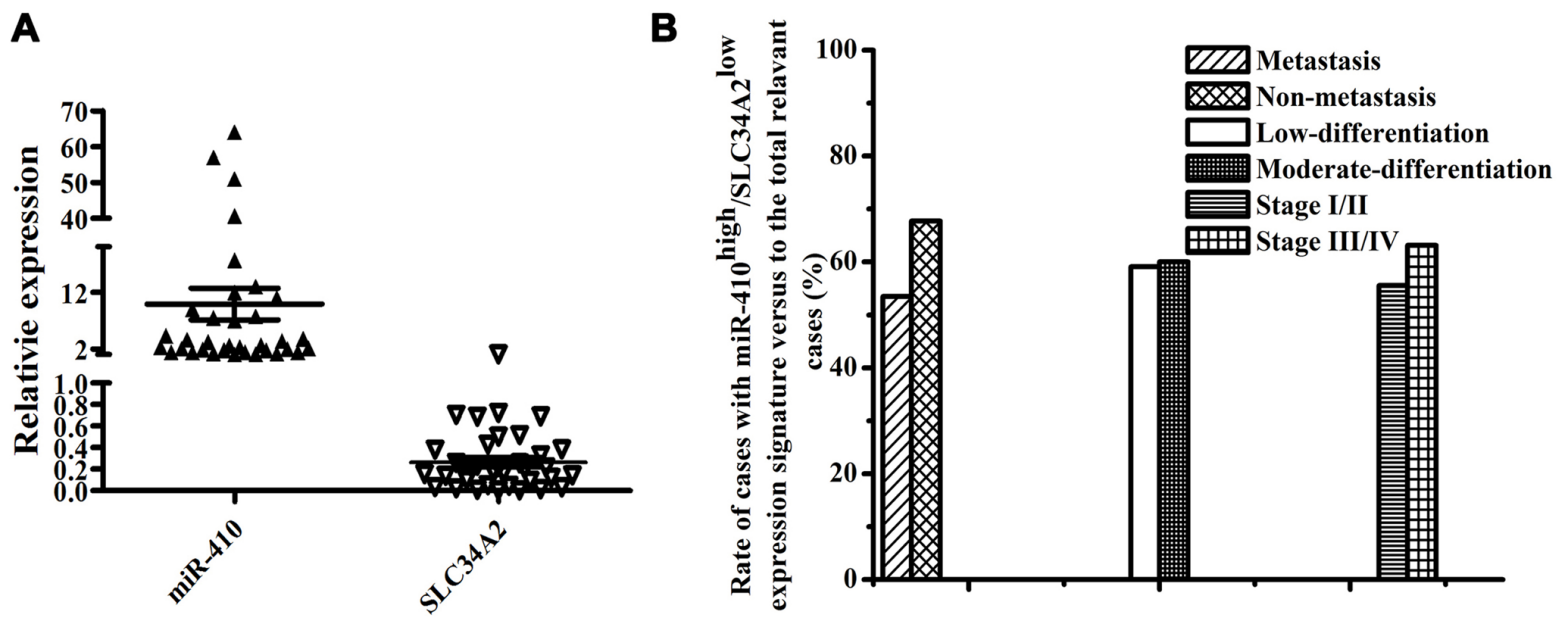
MiR-410 was highly expressed while SLC34A2 was lowly expressed in NSCLC tumor tissues **A.** The miR-410 expression was statistically higher while *SLC34A2* expression was considerably lower in 45 of 75 pairs of NSCLC tissues compared with their matched non-tumorous tissues. The expression level of miR-410 and *SLC34A2* were detected in 75 pairs of NSCLC frozen tissues and the adjacent non-tumorous tissues by qRT-PCR, and in 45 pairs of NSCLC tumor tissues that miR-410 expression levels were remarkably higher while *SLC34A2* expression levels were significantly lower. U6 small nuclear RNA and *β-actin* were used as internal control respectively. **B.** MiR-410^high^ /*SLC34A2*^low^ expression signature in NSCLC tumor tissues might not be correlated to the metastasis, differentiation or histopathological stage of NSCLC. *, *p* < 0.05; **, *p* < 0.01.

To determine the clinicopathological significance of the miR-410 and *SLC34A2* aberration, we evaluated the mRNA expression of miR-410 and *SLC34A2* in 75 pairs of frozen human NSCLC tumor tissues and adjacent non-tumorous lung tissues using qRT-PCR. MiR-410 or *SLC34A2* expression was not significantly associated with age and gender of the NSCLC patients (Table 1). However, miR-410 expression levels were remarkably higher while *SLC34A2* expression levels were significantly lower in 45 of 75 pairs of human NSCLC tumor tissues (45/75=60.0%) than that of their matched adjacent non-tumorous tissues respectively (Table 1, Figure 5A). Moreover, the rate of miR-410^high^/*SLC34A2*^low^ cases with tumor metastasis was lower than that of miR-410^high^/*SLC34A2*^low^ cases without tumor metastasis, while there was no statistical significance (*p* > 0.05) (Figure 5B). In addition, the rate of miR-410^high^/*SLC34A2*^low^ cases with low-differentiated tumors was not significantly different from that of miR-410^high^/*SLC34A2*^low^ cases with moderate-differentiated tumors (*p* > 0.05), and the rate of miR-410^high^/*SLC34A2*^low^ cases with tumors in stage I/II was also not significantly different from that of miR-410^high^/*SLC34A2*^low^ cases with tumors in stage III/IV (*p* > 0.05) (Figure 5B). Therefore, the expression of miR-410 and SLC34A2 were conversely correlated, and the miR-410^high^/*SLC34A2*^low^ expression signature frequently existed in human NSCLC tumor tissues but might not be correlated to the metastasis, differentiation or histopathological stage of NSCLC.

**Table 1:** Patient clinical features, miR-410 and SLC34A2 expressions profile

Patient No.	Gender	Age	Differentiation	Metastatic or non-metastatic	Histological grade	Clinical stage	Normalized miR-410 Expression in tumor tissues relative to adjacent non-tumorous tissues	Normalized SLC34A2 expression in tumor tissues relative to adjacent non-tumorous tissues
1	M	45	Medium	N	A	IIIB	1.755	0.7214
2	M	68	Low	Y	A	IIA	0.5587	2.1412
3	F	47	Low	Y	A	IIIB	0.6028	1.7791
4	M	51	Low	Y	A	IIIA	0.1763	1.4826
5	M	56	Low	N	A	IB	0.4247	0.7351
6	M	47	Low	N	A	IIA	7.4267	0.703
7	F	65	Low	Y	A	IIA	3.6209	0.2017
8	F	25	Low	Y	A	IV	1.1366	2.2838
9	F	71	Medium	Y	A	IIA	0.1811	0.561
10	M	67	Low	Y	A	IV	0.4827	1.9724
11	F	54	Low	N	A	IB	0.7007	1.523
12	F	72	Low	Y	A	IIIB	0.2345	1.351
13	M	43	Low	Y	A	IIB	1.3632	0.38
14	F	62	Low	Y	A	IIIA	1.1689	4.0905
15	F	48	Low	N	A	IIA	0.2972	2.7211
16	F	54	Low	Y	A	IIIA	51.0135	0.3841
17	F	53	Medium	N	A	IA	0.3602	0.1363
18	F	74	Low	Y	A	IIA	0.6011	0.6031
19	M	71	Low	N	A	IB	64.0105	0.2201
20	F	52	Low	N	A	IB	2.3203	0.0344
21	F	59	Low	Y	A	IIIB	1.2101	0.0516
22	F	79	Low	N	A	IB	17.6035	0.195
23	F	74	Low	N	A	IB	7.0731	0.6887
24	F	61	Low	Y	A	IIIA	13.0809	0.2298
25	F	69	Low	N	A	IIB	2.0741	0.1422
26	F	64	Medium	N	A	IB	57.0217	0.215
27	M	67	Low	N	A	IB	0.0711	0.2539
28	F	73	Low	Y	A	IIIB	1.4009	1.0359
29	M	64	Medium	N	A	IB	0.2662	1.5619
30	M	49	Low	Y	A	IIIA	1.7752	0.185
31	M	62	Medium-high	N	A	IIIA	0.8688	0.6332
32	M	73	Medium	Y	A	IIIA	0.0588	1.6946
33	F	70	Medium	Y	A	IIB	11.9298	0.2562
34	F	62	Medium	N	A	IIA	0.0783	5.3182
35	F	62	Medium-high	N	A	IIIA	0.8982	2.1794
36	F	28	Low	N	A	IIIA	1.4345	0.1355
37	F	57	Low	Y	A	IIB	0.5228	0.2823
38	M	61	Medium	N	A	IB	2.6379	0.0468
39	M	63	Medium	Y	A	IIA	0.1181	0.828
40	M	48	Low	Y	A	IIIA	1.3259	2.2234
41	M	41	Medium	N	S	III	0.4298	8.7846
42	M	57	Medium	N	S	IIB	0.2844	0.8287
43	M	59	Medium	N	S	IB	3.1964	0.0037
44	M	76	Low	N	S	IIIA	4.298	0.1547
45	M	54	Medium	N	S	IB	1.1705	0.3275
46	M	63	Medium	N	S	IIA	0.2511	0.1481
47	M	59	Medium	N	S	IIA	0.2843	0.0033
48	M	56	Medium	Y	S	IV	0.5287	0.1142
49	M	48	Medium	Y	S	IIIA	0.1802	0.4249
50	M	61	Medium	N	S	IB	0.3567	0.0092
51	M	49	Medium	N	S	IIIA	1.4163	0.0936
52	M	49	Low	Y	S	IIIA	0.3087	0.0031
53	M	62	Low	Y	S	IIIA	0.1055	0.2961
54	M	65	Low	Y	S	IIIA	0.0692	0.0185
55	M	55	Low	Y	S	IIIA	0.1646	0.024
56	M	44	Low	Y	S	IIA	0.7377	0.0359
57	M	66	Medium	N	S	IIIA	0.3227	0.0021
58	M	55	Low	Y	S	IIIA	1.0529	0.0142
59	M	50	Medium	Y	S	IIIA	3.3646	0.0067
60	M	53	Medium	Y	S	IIIA	2.5116	0.0308
61	M	53	Low	Y	S	IIA	0.0246	0.7127
62	M	50	Low	N	S	IB	1.1022	0.0051
63	M	74	Low	N	S	IA	1.8755	0.0982
64	M	58	Low	Y	S	IIIA	0.0394	0.2299
65	M	55	Medium	Y	S	IIIA	2.3041	0.255
66	M	41	Low	Y	S	IIIA	9.0098	0.5008
67	M	40	Medium	Y	S	IIIB	2.0088	0.4269
68	M	63	Medium	Y	S	IIIA	0.1397	0.6806
69	M	56	Low	Y	S	IIB	0.0021	0.0075
70	M	65	Medium	Y	S	IIIA	40.6273	0.1683
71	M	66	Low	Y	S	IIA	7.7249	0.0222
72	M	58	Low	Y	S	IIIA	11.0334	0.1197
73	M	57	Low	Y	S	IIIA	3.7474	0.6882
74	M	62	Medium	Y	S	IIB	0.6636	0.8524
75	M	62	N/A	N/A	N/A	N/A	2.1138	0.5149

Note: M, male; F, female; A, adenocarcinoma; S, squamous cell carcinoma; L, large cell carcinoma; Y, yes; N, no. Relative expression of miR-410 and SLC34A2 were performed by the 2^−ΔΔCt^ method with adjacent non-tumorous lung tissues as a calibrator. Data show the means from independent analyses. Every independent analysis by Real - time PCR was carried out immediately after the RNA extraction and reverse transcribed. ΔCt obtained from real-time PCR was subjected to paired t-test (ΔCt=Ct_SLC34A2_-Ct_β-actin_).

### MiR-410 functioned as oncogene by downregulating *SLC34A2 via* activating Wnt/β-*catenin* pathway in NSCLC cells

**Figure 6:**
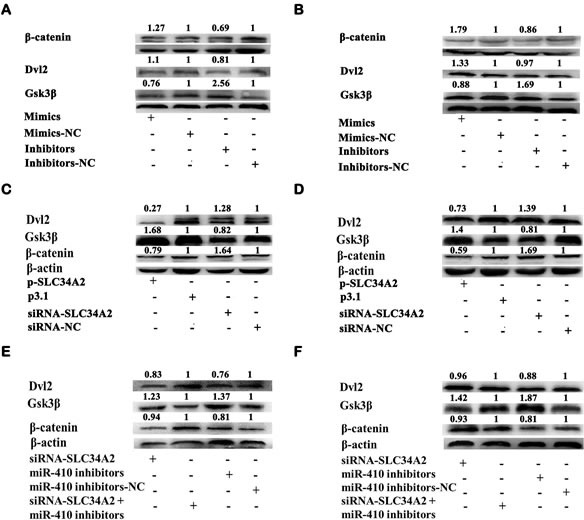
MiR-410 functioned by targeting SLC34A2 through Wnt/β-catenin pathway in NSCLC cells (A and B) Western blotting analysis of *β-catenin*, *DVL2* and *Gsk3β* protein expression in A549 **A.** and 95D **B.** cells after transfecting with miR-410 inhibitors/NC or mimics/NC. Overexpression of miR-410 significantly up-regulated *DVL2* and *β-catenin* respectively, and down-regulated *Gsk3β*, while inhibition of miR-410 down-regulated *DVL2* and *β-catenin* respectively, and up-regulated *Gsk3β* in both A549 **A.** and 95D **B.** cells compared with their respective control. (C and D) Western blotting analysis of *β-catenin*, *DVL2* and *Gsk3β* protein expression in A549 **C.** and 95D **D.** cells after cotransfecting with p3.1-*SLC34A2/*p3.1 or siRNA-*SLC34A2*/siRNA-NC. Overexpression of *SLC34A2* significantly down-regulated *DVL2* and *β-catenin* respectively, and up-regulated *Gsk3β*, while inhibition of *SLC34A2* up-regulated *DVL2* and *β-catenin* respectively, and down-regulated *Gsk3β* in both A549 **C.** and 95D **D.** cells compared with their respective control. (E and F) Western blotting analysis of *β-catenin*, *DVL2* and *Gsk3β* protein expression in A549 **E.** and 95D **F.** cells after cotransfecting with miR-410 inhibitors and siRNA-*SLC34A2*. Down-regulation of *DVL2* and *β-catenin*, and up-regulation of *Gsk3β* were partly impaired in both A549 **E.** and 95D **F.** cells after cotransfecting with miR-410 inhibitors/NC and siRNA-*SLC34A2* compared with that of cells transfecting with miR-410 inhibitors/NC. *, *p* < 0.05; **, *p* < 0.01, significant difference vs NC control.

Wnt/*β-catenin* pathway played a significant role in lung cancer tumorgenesis. To explore the mechanism of how miR-410 functioning via targeting SLC34A2 in NSCLC cells, miR-410 inhibitors/NC or miR-410 mimics/NC were firstly transfected into A549 and 95D cells and western blotting was performed to detect the change of core protein levels of Wnt/*β-catenin* pathway. We found that overexpression of miR-410 significantly up-regulated the protein expression of *DVL2* (1.1 fold and 1.33 fold respectively) and *β-catenin* (1.27 fold and 1.79 fold respectively), and down-regulated *Gsk3β* (0.76 fold and 0.88 fold respectively), while inhibition of miR-410 significantly down-regulated the protein expression of *DVL2* (0.81 fold and 0.97 fold respectively) and *β-catenin* (0.69 fold and 0.86 fold respectively), and up-regulated *Gsk3β* (2.56 fold and 1.69 fold respectively) in both A549 (Figure 6A) and 95D (Figure 6B) cells. Then, we detected the effect of *SLC34A2* on the core protein levels of Wnt/*β-catenin* pathway after transfecting with p3.1-*SLC34A2*/p3.1 or siRNA-*SLC34A2*/siRNA-NC in A549 and 95D cells by western blotting. Conversely, overexpression of *SLC34A2* significantly down-regulated the protein expression of *DVL2* (0.27 fold and 0.73 fold respectively) and *β-catenin* (0.79 fold and 0.59 fold respectively), and up-regulated *Gsk3β* (1.67 fold and 1.4 fold respectively), while inhibition of *SLC34A2* significantly up-regulated the protein expression of *DVL2* (1.28 fold and 1.39 fold respectively) and *β-catenin* (1.64 fold and 1.69 fold respectively), and down-regulated *Gsk3β* (0.82 fold and 0.81 fold respectively) in both A549 (Figure 6C) and 95D (Figure 6D) cells. These data testified that the function of miR-410 and *SLC34A2* in NSCLC cells were correlated to influencing Wnt/*β-catenin* pathway.

Given that miR-410 directly targeted *SLC34A2* and inhibition of miR-410 prohibited the Wnt/*β-catenin* pathway, we next tested whether *SLC34A2* interference could rescue the inhibition of Wnt/*β-catenin* pathway by miR-410 interference in NSCLC cells. We found that down-regulation of *DVL2* (0.76 fold and 0.88 fold respectively) were increased to 0.83 fold and 0.96 fold respectively, and down-regulation of *β-catenin* (0.81 fold and 0.79 fold respectively) were increased to 0.94 fold and 0.93 fold respectively, and up-regulation of *Gsk3β* (1.37 fold and 1.87 fold respectively) were decreased to 1.23 fold and 1.42 fold respectively in both A549 (Figure 6E) and 95D (Figure 6F) cells after co-transfecting with miR-410 inhibitors and siRNA-*SLC34A2* compared with that of transfecting with miR-410 inhibitors. These results validated that the molecular mechanism of miR-410 acting as oncogene through down-regulating *SLC34A2* was correlated to Wnt/*β-catenin* pathway.

### Abnormal expressions of miR-410 might be regulated neither by DNA methylation nor by deacetylation in NSCLC cells

**Figure 7:**
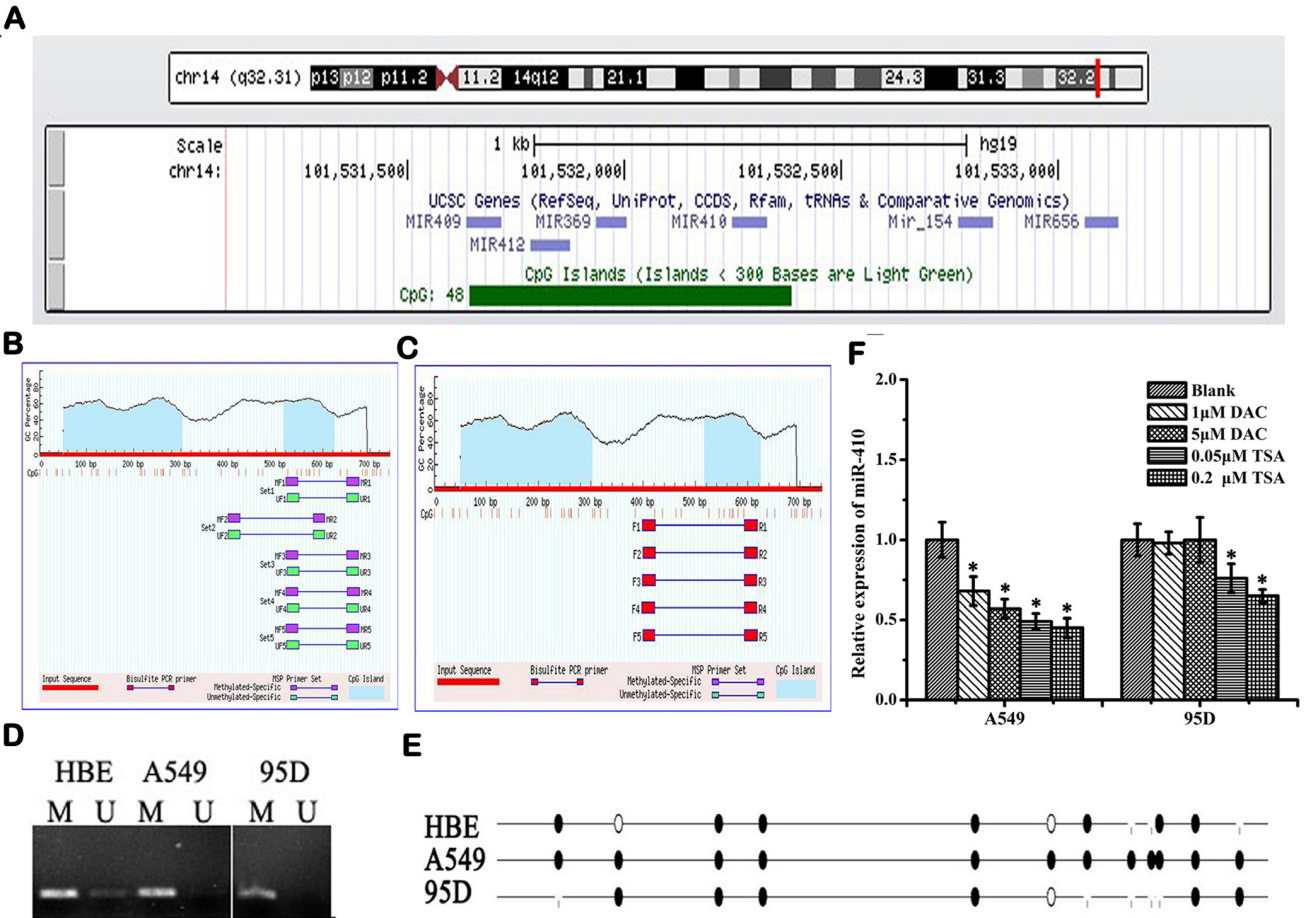
Abnormal expression of miR-410 is not regulated by methylation or acetylation in NSCLC cells **A.** CpG island located in the gene locus of miR-410 according to UCSC database (http://genome.ucsc.edu/cgi-bin/hgGateway). Blue areas: predicted CpG islands (Locus: chr14: 101531644-101532384, GC content 67.2%, Obs/Exp value 0.8). (B and C) Primers were designed to cover the CpG islands upward miR-410 for methylation analysis. Primer sets for MSP **B.** and BSP **C.** according to MethPrimer (http://www.urogene.org/methprimer/index1.html). **D.** MSP results detected by 2% agarose gel electrophoresis in A549, 95D and HBE cells. U, unmethylated; M, methylated. HBE, human bronchial epithelial cells. The result indicated that miR-410 was partly methylated in normal HBE and 95D cells, but almost totally methylated in A549 cells. **E.** Bisulfite sequencing analysis of miR-410 in A549, 95D and HBE cells. Black dot, methylated; White dot, unmethylated. The result of bisulfite sequencing (BSP) showed that all the 12 CpGs sites sequenced were completely methylated in A549 cells, and 6 of 12 CpGs sites (50%) were methylated in 95D cells, and 7 of 12 CpGs sites (58.3%) were methylated in HBE cells, in consistent with the result of MSP. **F.** Expression levels of miR-410 were detected by qRT-PCR in A549 and 95D cells after treatment with DNA methylation inhibitor 5′-aza-2′-deoxycytidine (DAC) and/or histone deacetylase (HDAC) inhibitor trichostatin A (TSA). Data are expressed as means ± SD of triplicate assays. *, *p* < 0.05; **, *p* < 0.01, significant difference vs untreated group.

The genomic locus embedding miR-410 is surrounded by a CpG island (741 bps, CG content 57.2%, Obs/Exp value 0.8) according to the Database UCSC (Figure 7A). In order to determine whether miR-410 abnormal expression was regulated by CpG methylation, we firstly designed several primer pairs for methylation analysis by methylation specific PCR (MSP) in A549 and 95D cells (Figure 7B and 7C). The MSP result indicated that miR-410 was partly methylated in normal HBE and 95D cells, but almost totally methylated in A549 cells (Figure 7D). Moreover, the result of bisulfite sequencing (BSP) showed that all the 12 CpGs sites sequenced were completely methylated in A549 cells, and 6 of 12 CpGs sites (50%) were methylated in 95D cells, and 7 of 12 CpGs sites (58.3%) were methylated in HBE cells, in consistent with the result of MSP (Figure 7E). However, down-regulation expression of miR-410 were detected in A549 cells and 95D cells treated with DNA methylation inhibitor 5′-aza-2′-deoxycytidine (DAC) (Figure 7F). It suggested that miR-410 was not silenced by DNA methylation in A549 and 95D cells. Similarly, down-regulation expression of miR-410 was also detected in NSCLC cells treated with histone deacetylase inhibitor trichostatin A (TSA) (Figure 7F). It suggested that histone deacetylation might not be involved in regulating miR-410 expression.

## DISCUSSION

In this study, we identified that miR-410 directly targeted the 3′UTR of *SLC34A2* and suppressed its expression transcriptionally and post-transcriptionally, and confirmed that miR-410 promoted the proliferation, invasion and migration of NSCLC cells by down-regulating *SLC34A2 in vitro and in vivo*. And we revealed miR-410^high^ /*SLC34A2*^low^ expression signature frequently existed in human NSCLC tumor tissues. We also demonstrated that miR-410 functioned as oncogene by down-regulating *SLC34A2* expression via activating Wnt/*β-catenin* pathway.

Recent studies reported that miR-410 affected many physiological cellular processes, such as gene expression, cell proliferation, migration and invasion, etc., and might act as either tumor promoter or tumor suppressor and associated with malignant phenotypes [29-32]. Herein, our data indicated that miR-410 promoted cell proliferation, invasion and migration of NSCLC cells *in vitro* and *in vivo*. Therefore, miR-410 appealed to be an oncogene in NSCLC. Our results were consistent with the reports that miR-410 negatively regulated pRb/E2F pathway by directly targeting CDK1 and was an oncogene in breast cancer [29], and that miR-410 was highly expressed in liver and colorectal tumors, and enhanced tumor cell growth by silencing FHL1 and thus served as oncomiR [33]. However, our results were converse with the reports that miR-410 was down-regulated in human gliomas. Overexpression of miR-410 in glioma cells strongly inhibited cell proliferation and invasion mediated by targeting MET [30], and that miR-410 directly targeted VEGF and inhibited cell proliferation and contributed to apoptosis in osteosarcoma cells [31], and that miR-410 was lowly expressed in gastric cancer and suppressed migration and invasion by targeting MDM2 [32]. It is likely that the discrepancy was caused by that their observations were attained in different type of tumors which have varied clinical or pathological features with that used in our study.

*SLC34A2* is expressed on cell surfaces as a heavily glycosylated plasma membrane protein for mediating the transport of inorganic phosphate into epithelial cells via sodium ion co-transport [6, 7, 34]. Increased inorganic polyphosphate has been reported to promote the proliferation of human fibroblasts and human dental pulp cells [35]. This transporter has also been considered to be associated with cell differentiation [36] and may play a role in tumorigenesis [15]. However, the functional analyses of *SLC34A2* in tumorigenesis have yielded contradictory results in different cancer models. *SLC34A2* was highly expressed in thyroid cancer and breast cancer [3], but lowly expressed in non-small cell lung carcinomas tissues [15]. In this study, we demonstrated that *SLC34A2* was directly targeted by miR-410 and inhibited by miR-410 transcriptionally and post-transcriptionally, and the biological roles of miR-410 in NSCLC were mediated by down-regulating *SLC34A2 in vitro* and *in vivo*, indirectly implying *SLC34A2* played as tumor suppressor in NSCLC. We showed *in vitro* that knockdown of *SLC34A2* blocked the effect of miR-410 inhibition, which were critical to tumor cell biology, namely, proliferation, migration and invasion. In addition, the expression of miR-410 was negatively correlated with that of *SLC34A2* in human NSCLC tumor tissues. It seemed that miR-410^high^/*SLC34A2*^low^ expression signature frequently existed in NSCLC cells and patient tumor tissues, while was not statistically correlated to the clinical or pathological feature of tumor tissues. Moreover, miR-410 or *SLC34A2* alone was not statistically correlated to the clinical or pathological feature of tumor tissues as well (Data not shown). In the future, we will collect more human NSCLC tumor tissues to further statistically analyze the relationship between miR-410^high^/*SLC34A2*^low^ expression signature with the histopathological features.

Wnt/β-catenin signalling was involved in a wealth of developmental processes and the maintenance of adult tissue homeostasis by regulating cell proliferation, differentiation, migration, genetic stability and apoptosis, as well as by maintaining adult stem cells in a pluripotent state [37]. Aberrant regulation of this pathway was therefore associated with a variety of diseases, including cancer, fibrosis, et al [37]. Recent study in rats muscle cells found that miR-410 could inhibit the sFRP (Secreted frizzled-related proteins, an endogenous modulator of Wnt signaling that compete with the Wnt ligands for the binding to the Frizzled receptors expression) thereby activating Wnt/*β-catenin* signaling pathway [38]. Our data showed miR-410 played a reverse role in regulating the expression of DVL2, β-catenin and Gsk3β compared with *SLC34A2*. And up-regulation of DVL2 and β-catenin and down-regulation of Gsk3β by miR-410 were partly rescued by *SLC34A2*. It indicated that miR-410 activated Wnt/β-catenin signaling pathway via down-regulating *SLC34A2* in NSCLC.

So far, the molecular mechanism of abnormal expression of miR-410 was poorly understood. In recent years, more and more studies showed that miRNA was regulated by epigenetic mechanisms [39]. As the main mechanisms of epigenetic, methylation and histone modifications have been shown to synergistically regulate gene expression [39]. Methylation occurs mainly in CpG islands of gene promoter. In a variety of tumors, methylation status of CpG islands in miRNA promoter was closely related to its expression and loss of function [40]. Many miRNAs which acted as tumor suppressor were inactivated because of high hypermethylation in tumor cells or tissues, while those of oncogenic miRNAs were activated for its hypomethylation [40]. In present experiment, we found an CpG islands in upstream of miR-410 promoter region (Locus: chr14: 101531644-101532384, GC content 67.2%, Obs/Exp value 0.8) through UCSC database, and it was hypermethylated in A549 and 95D cells compared with normal human bronchial epithelial cells through methylation-specific PCR and bisulfite sequencing. However, methylation inhibitor DAC (5-aza-2′-deoxycytidine) or histone deacetylase inhibitor TSA (Trichostatin A) treatment failed to increase miR-410 expression in both A549 and 95D cells, indicating that methylation and acetylation did not modulate the expression of miR-410. Therefore, we inferred some other unknown factors but methylation or deacetylation were involved in regulating the expression of miR-410, such as specific transcription factor or long non-coding RNA or some other activating factors etc. In the next plan, we will strive to use comprehensive approaches, such as transcription factor or miRNA epigenome arrays etc. to further elucidate its regulating mechanism.

In conclusion, miR-410 could act as oncogene in the development and progression of NSCLC by down-regulating *SLC34A2* via activating Wnt/*β-catenin* signaling pathway. With more understanding its function, miR-410 may be used as a potential therapeutic target for NSCLC.

## MATERIALS AND METHODS

### MiRNA target prediction by bioinformatics methods

The miRNA targets predicted by publicly available algorithms were obtained from miRanda (http://www.microrna.org/microrna/home.do) and TargetScan (http://www.targetscan.org). Putative target genes predicted by both algorithms were accepted.

### Cell culture and animals

HBE, A549, H1299 and 293 cell lines were from American Type Culture Collection (USA). 95D cell lines were purchased from Shanghai Institute of Cell Bank (Shanghai, China). The A549, 95D and H1299 cell lines were cultured in RPMI 1640 (Invitrogen, Carslabd/CA, USA), and HBE/HEK293 cell lines were cultured in dulbecco's modified eagle medium (DMEM) (Invitrogen, Carslabd/CA, USA) supplemented with 10% fetal bovine serum (Invitrogen, Carslabd/CA, USA) at 37°C in 5% CO_2_. Female athymic BALB/c nude mice, 3-4 weeks old, obtained from HFK Bioscience (Beijing, China), were maintained at the Animal Core Facility at West China Hospital, Sichuan University under specific pathogen-free (SPF) condition. All studies on mice were conducted in accordance with the National Institutes of Health ‘Guide for the Care and Use of Laboratory Animals’.

### Lung tumor patient samples

NSCLC tumor and normal adjacent NT tissue specimens were obtained from 75 patients from Department of Thoracic Surgery, West China Hospital, Sichuan University. This study was performed with the approval of the Medical Ethical Committee of West China Hospital, Sichuan University. A summary of the patients tumor sample characteristics were shown in Table 1.

### RNA extraction and quantitative real-time PCR

Total RNA of NSCLC cell lines and patient tissues were isolated with TRIzol (Invitrogen, Carslabd/CA, USA) according the manufacturer's instructions. cDNAs were generated using PrimeScript™ RT-PCR Kit (Takara Biotech (Dalian) Co., Ltd, Dalian, China). Primers for qRT-PCR were as follows: *SLC34A2* (NM_006424): Forward, 5′- GAG AAC ATC GCC AAA TGC-3′; Reverse, 5′- GCA ACC ACA GAG GAC CAG-3′. *β-actin*: Forward, 5′-CTT AGT TGC GTT ACA CCC TTT CTTG-3′; Reverse, 5′-CTT AGT TGC GTT ACA CCC TTT CTTG-3′. MiR-410 (MIMAT0002171) stem-loop primers and U6 primers were commercially synthesized (RiboBio Co., Ltd, Guangzhou, China). QRT-PCR was performed using a SYBR Green Real-time PCR Master Mix Kit protocol (Bio-Rad, Hercules/CA, USA) on CFX96 Real-Time System (Bio-Rad, Hercules/CA, USA). *β-actin* and U6 were used as internal controls for *SLC34A2* and miR-410 respectively. Relative quantification of miR-410 or *SLC34A2* expression was calculated using the 2^−ΔΔCt^ method. All reactions were done in triplicate.

### Construction of reporter plasmids and luciferase reporter assay

Dual-luciferase reporter system (Promega (Beijing) Biotech Co., Ltd, Beijing, China) was used to analysis whether miR-410 would directly target the 3′UTR of *SLC34A2*. The 3′UTR sequence of the *SLC34A2* was amplified by PCR using the flowing primers: Forward, 5′-GCG AGC TCG CTG CGC TCC AGC CTT ATCT-3′; Reverse, 5′-GCT CTA GAG CAA GCC TGC CTC ATT TCC A-3′, and the ~200 bp amplicon was cloned into pMIR vector (Promega (Beijing) Biotech Co., Ltd, Beijing, China) to produce wild-type reporter (PmirGLO- *SLC34A2* 3′UTR-F). The mutant reporter (PmirGLO-*SLC34A2* 3′UTR-R) construct was generated by fusing the reversed amplicon (amplified by using the flowing primers: Forward, 5′-GCG CTA GCG CCC ACA GAT GGG CTT TGAT-3′; Reverse, 5′-GCT CTA GAG CCT TGC TGC ACG GCT ACAC-3′) into pMIR vector. For luciferase reporter assay, HEK-293 cells cultured in 96-well plate were cotransfected with PmirGLO-*SLC34A2* 3′UTR-F or PmirGLO-*SLC34A2* 3′UTR-R, and miR-410 mimics or negative control (NC), following the manufacturer's protocol of Lipo2000 (Invitrogen, Carslabd/CA, USA). Luciferase activity was detected 24h post-transfection according to dual-luciferase reporter assay system (Promega (Beijing) Biotech Co., Ltd, Beijing, China). The Renilla luciferase signals were normalized to the internal firefly luciferase transfection control. Transfections were done at least thrice in independent experiments.

### Western blotting

Western Blotting was used to detect the influence of miR-410 and *SLC34A2* on key proteins of Wnt/*β-catenin* signaling pathway. A549 or 95D cells were transfected with miR-410 mimics/NC or miR-410 inhibitors/NC, or transfected with p3.1-*SLC34A2*/p3.1 or siRNA-*SLC34A2*/siRNA-NC or cotransfected with siRNA-*SLC34A2* and miR-410 inhibitors/NC according to the instructions of Lipo2000. The total membrane proteins were extracted 24hr post-transfection according to the manufacturer's instructions (Promega (Beijing) Biotech Co., Ltd, Beijing, China). Total cell protein was extracted using RIPA lysis buffer containing protease inhibitor cocktail at 1:100 dilution. Protein concentrations were measured using a BCA protein assay kit. The protein level was quantified by Western blotting analysis of 50 μg of cell extracts or tissue extracts. The following primary antibodies were used: anti-*β-catenin* (Cell Signaling Technology, Danvers, MA, USA, 1:1000), anti-*DVL2* (Cell Signaling Technology, Danvers, MA, USA, 1:1000), anti-*Gsk3β* (Cell Signaling Technology, Danvers, MA, USA, 1:1000), anti-*β-actin* (Cell Signaling Technology, Danvers, MA, USA, 1:1000), anti-*SLC34A2* (Santa Cruz, CA, USA, 1:500), *β-actin* was used as an internal control.

### Cell proliferation assay

*In vitro* proliferation assay was used to detect the influence of miR-410 on the growth of A549 and 95D cell lines and also determine whether *SLC34A2* were engaged in miR-410 inhibitor-mediated growth suppression. A549 or 95D cells cultured in 96-well plates (4000/well) were transfected with miR-410 inhibitors/NC or miR-410 mimics/NC, or cotransfected with miR-410 inhibitors/NC and siRNA-*SLC34A2/*NC. Cell viability was evaluated with 3-(4, 5-dimethylthiazol-2-yl)-2, 5-diphenyltetrazolium bromide (MTT; Sigma, St. Louis, MO, USA) as described previously [26].

### Flow-cytometric analysis of apoptosis

In order to detect the influence of miR-410 on apoptosis of A549 and 95D cells. Cells cultured in 6-well plates were transfected with miR-410 inhibitors/NC or miR-410 mimics/NC. 24h after transfection, cells (2×10^5^ /well) were seeded into fresh six-well plates. FITC-Annexin V and Propidium iodide (PI) was added 24h later and apoptotic cells were identified with a flow cytometer (BD Biosciences, San Diego, CA, USA) according to manufacturer's protocol.

### *In vitro* invasion and migration assay

*In vitro* Transwell and Millicell assay were used for detecting the influence of miR-410 on the invasion and migration of A549 and 95D cell lines by targeting *SLC34A2*. A549 or 95D cells cultured in 6-well plates were transfected with miR-410 inhibitors/NC or miR-410 mimics/NC, or cotransfected with miR-410 inhibitors/NC and siRNA-*SLC34A2* respectively. For the migration assays, cell dilutions in serum-free media were placed respectively into the upper chamber of an insert (8-μm pore size, Millipore, Billerica, MA, USA). For the invasion assays, A549 or 95D cells cultured in 6-well plates were transfected with miR-410 inhibitors/NC or miR-410 mimics/NC, or cotransfected with miR-410 inhibitors /NC and siRNA-*SLC34A2* respectively. 24h after transfection, cells were harvested and cell dilutions were placed into the upper chamber of an insert coated with Matrigel (BD Biosciences, San Diego, CA, USA). Media containing 10% FBS were added to the lower chamber. After 24 hours of incubation, the cells remaining on the upper membrane were removed with cotton wool, whereas the migrated or invaded cells were stained with methanol and 0.1% crystal violet, then were imaged and counted.

### Establishment of miR-410 knockdown stable cell line

MiR-410 inhibitor recombinant lentivirus solution (HmiR-AN0244-AM03, Guangzhou Fulengen Co., Ltd, Guangzhou, China) and control lentivirus solution (CmiR-AN0001-AM03, Guangzhou Fulengen Co., Ltd, Guangzhou, China) were purchased from company. For generation of miR-410 knockdown stable cell line (INH-LV) and negative control (INH-NC-LV), A549 cells were infected with lentivirus particles containing miR-410 inhibitor vector (HmiR-AN0244-AM03, Guangzhou Fulengen Co., Ltd, Guangzhou, China) or scrambled control clone for pEZX-AM03 (CmiR-AN0001-AM03, Guangzhou Fulengen Co., Ltd, Guangzhou, China) with Polybrene (5 μg/ml; Sigma, St.Louis, MO, USA). Medium containing lentiviral particles was replaced with fresh medium 24 h post-infection and then incubated for 72 h. Stable cells were selected after infection using hygromycin (200μg/ml; Roche, USA) by 4 weeks. Medium containing hygromycin was replaced every 3 days. The stable cell lines were further identified by detection of miR-410 expression by qRT-PCR.

### Animal study

In order to detect the effects of miR-410 on migration and invasion of non-small lung cancer cells *in vivo*, miR-410 knockdown stable cells INH-LV and negative control INH-NC-LV cells were used to establish lung metastasis mouse model respectively according to the described method [28, 29]. Briefly, cells were injected via tail vein into 3 week-old BALB/c nude mice (3×10^6^ cells per mouse, eight mice in each group). About five weeks later, eight mice were anesthetized, lungs of five mice were injected intratracheally with India ink and fixed by AAF solution (85% ethanol, 10% acetic acid, 5% formalin) to count the number of metastatic tumor nodules (white dots) on lung surfaces. The sizes of the metastatic nodules were observed and the relative metastatic inhibition ratio was calculated in terms of the tumor nodules in INH-LV and INH-NC-LV group relative to that of blank control. Then, lungs of three mice were excised and fixed in 4% buffered paraformaldehyde for further pathological analysis. Lung sections were stained with hematoxylin and eosin (H&E) to visualize the metastatic tumor nodules in lungs.

### Methylation-specific PCR (MSP) and bisulfite sequencing

In order to determine whether miR-410 abnormal expression was regulated by CpG methylation, we checked the methylation status of CpG islands 48 upstream of miR-410 in NSCLC cell lines. Genomic DNA was extracted using Universal Genomic DNA Extraction Kit Ver.3.0 (Takara Biotech (Dalian) Co., Ltd, Dalian, China). Sodium bisulfite modification was applied to the genomic DNA in line with the CpGenome™ Fast DNA Modification Kit (Chemicon, Billerica, MA, USA).

Methylation-specific PCR and bisulfite sequencing were used to detect the methylation status of CpG islands 48 of miR-410. Firstly, genomic DNA after bisulfite modification was amplified according to the instructions of EpiTect Whole Bisulfitome Kit (Qiagen, Valencia, CA, USA). Then 2 μL of the amplified products were used as templates for methylation specific PCR (MSP) and bisulfite sequencing PCR (BSP). MethPrimer (http://www.urogene.org/methprimer/index.html) software were used to design MSP and BSP primers and the MSP primer sequences were as follows: Forward-methylated: 5′-GTT TTT TTG AGG GTA GGA GTA GGA C-3′; Reverse-methylated: 5′-AAA TAC CAT CGA CTC AAA AAC GTA-3′; Forward-unmethylated: 5′-GTT TTT TTG AGG GTA GGA GTA GGA T-3′; Reverse-unmethylated: 5′-AAA TAC CAT CAA CTC AAA AAC ATA-3′. Primer sequences for BSP were as follows: Forward, 5′-AGT TTT TTT GAG GGT AGG AGT AGG A-3′; Reverse, 5′-CTT CTC AAA TAC CCA AAA TAC CAT C-3′. BSP products from gel recycling were cloned into pGM-T vector according to the manufacturer's instructions (Tiangen Biotech Co., Ltd, Beijing, China) and transformed into DH5α competent cells. At least five positive clones in each cell line or control were picked for sequencing.

To further detect the effects of methylation or acetylation on miR-410 expression, methylation inhibitor DAC (5′-aza-2′-deoxycytidine; Sigma, St.Louis, MO, USA) and histone deacetylase inhibitor TSA (trichostatin A; Sigma, St.Louis, MO, USA) were used to treat cells. 95D and A549 cells (3×10^5^/well) seeded in 6-well plates were treated with DAC (1 or 5 μM, or TSA (0.05 or 0.2 μM) for 24h respectively. Cells were harvested after treatment and total mRNA was extracted for detecting the expression of miR-410 by qRT-PCR.

### Statistical analysis

The data were presented as mean ± SD of at least three independent experiments. The significance of difference between the experimental groups and control was assessed by one-way ANOVA analysis in SPSS 19.0 software. A value of *p* < 0.05 was considered as statistically significant.

## Abbreviations

NSCLC: non-small cell lung cancer
QRT-PCR: quantitative real-time PCR
3′UTR: 3′untranslated region
miRNA: microRNA
mRNA: messenger RNA
